# The impact of poor oral health on the oral health-related quality of life (OHRQoL) in older adults: the oral health status through a latent class analysis

**DOI:** 10.1186/s12903-019-0840-3

**Published:** 2019-07-10

**Authors:** Lyzbeth Beatriz Ortíz-Barrios, Víctor Granados-García, Pablo Cruz-Hervert, Karla Moreno-Tamayo, Erika Heredia-Ponce, Sergio Sánchez-García

**Affiliations:** 1 Unidad de Investigación Epidemiológica y en Servicios de Salud, Área Envejecimiento, Centro Médico Nacional Siglo XXI, Instituto Mexicano del Seguro Social, Av. Cuauhtémoc No. 330, Edificio CORCE, 3er piso, Col. Doctores, Delegación Cuauhtémoc, CP 06720 Mexico City, Mexico; 2 División de Estudios de Posgrado e Investigación en Odontología, Universidad Nacional Autónoma de México, Mexico City, Mexico; 3 Departamento de Salud Pública y Epidemiología Bucal, Facultad de Odontología, Universidad Nacional Autónoma de México, Mexico City, Mexico

**Keywords:** Geriatrics, Oral health, Quality of life, Latent class analysis

## Abstract

**Background:**

Determine the impact of poor oral health on the oral health-related quality of life (OHRQoL) in community-dwelling older adults.

**Methods:**

Cross-sectional study of community-dwelling older adults in Mexico City. Sociodemographic characteristics were obtained and assessed their OHRQoL according to the Geriatric/General Oral Health Assessment Index (GOHAI). Clinical evaluation of their oral health: painful chewing, use of dentures, dry mouth, xerostomia, plaque, calculus, coronal and root caries, tooth loss and gingival bleeding. Finally, we determined the oral health of participants through Latent Class Analysis (LCA), excluding totally edentulous. The strength of association was determined (Odds Ratio [OR] and 95% confidence interval [95% CI]) through logical regression between the oral health categories (latent classes) and OHRoL in older adults, adjusted with the other variables included in the study: age, sex, marital status, living arrangements (lives alone), educational level, paid work status, comorbidity, cognitive deterioration, depression and use of medical and dental services in the previous 12 months.

**Results:**

The mean (SD) GOHAI score for the 228 older adults to 46.5 (8.7), number of classes to characterize oral health through LCA was three (entropy 0.805). The GOHAI mean for Class 3 (57.0%), acceptable oral health was 50.1 (7.1); totally edentulous (9.6%), 47.9 (8.4); for Class 2 (16.7%), regular oral health, 43.8 (9.3); and for Class 1 (16.7%), poor oral health, 42.2 (9.7). Significant differences were observed among means (*p* < .001). Using Class 3 an as a reference, the strength of association between the GOHAI scores and low OHRQoL (GOHAI 25th percentile = 24.0) was OR = 0.7, 95% CI = 0.2–3.3 for totally edentulous; OR = 3.0, 95% CI = 1.2–7.6 for Class 2 and OR = 5.0, 95% CI = 2.1–12.1 for Class 1.

**Conclusion:**

Poor oral health was associated with a negative impact on the OHRQoL of community-dwelling older adults.

**Clinical relevance:**

It is essential to design and implement oral health care policies specifically targeted at improving the quality of life in this older adult population.

## Background

Research on the oral health of older adults has expanded over the past decades with the continuous growth of this population sector worldwide [1]. While today, older adults keep their teeth longer, they suffer from poor oral health because of accumulated deficits in their oral cavity. The reason that older adults lose their teeth is not age but the burden of inadequately controlled chronic diseases and poor oral hygiene. This is compounded by the fact that they seek oral health services less frequently than does the rest of the population [1–3].

Methods for estimating oral health deficits in older adults have traditionally relied on oral indices and clinical indicators. However, those methods are seriously limited, primarily because they report the presence/absence of diseases separately and classify individuals according to patterns defined exclusively on the bases of their oral health deficits [4]. The purpose of using the latent class analysis (LCA) in this study was to distinguish the optimal number of classes that characterize the oral health of older adults. The LCA is an approach that classifies individuals into groups based on the patterns of individual response.

Although the aging process affects all dimensions of life, including personal wellbeing, few studies have explored the impact of oral health on the different dimensions of quality of life in older adults [5].

Subjective indicators have been employed to estimate the extent to which oral health deficits affect physical functioning and psychosocial wellbeing [6, 7]. Salient among them, the Geriatric/General Oral Health Assessment Index (GOHAI) has been widely used to estimate the oral health-related quality of life (OHRQoL) [8].

GOHAI is based on three suppositions: a) that oral health can be measured using self-evaluation; b) that the levels of oral health vary among people and that this variation can be demonstrated using measurement based on a person’s self-perception; and c) that self–perception has been identified as predictive of oral health. The GOHAI measures respondents’ oral functional problems in a compact questionnaire of 12 items that, in a simply administered manner, evaluate problems related to oral health in the past 3 months. It is also designed to estimate the severity of psychosocial impacts associated with oral diseases. Instrumental in assessing needs and levels of satisfaction, the GOHAI is a valuable resource for evaluating the results of oral health programs and services. The resulting evidence, in turn, provides the bases for designing effective policies for the overall or specific sectors of the population [7, 8].

Understanding the impact of poor oral health on the different dimensions of quality of life enables us to identify areas of opportunity for programs and policies seeking to improve the health status of the population. The aim of this study was to determine the impact of poor oral health on the OHRQoL in community-dwelling older adults.

## Methods

### Sample and recruitment

From June to August 2014, we conducted a cross-sectional study of a representative sample of older adults affiliated to a Family Medicine Unit (FMU) operated by the Mexican Institute of Social Security (IMSS by its Spanish initials) in southeastern Mexico City. The sample size was calculated under the assumption that 15.2% of older adults lose all of their teeth (total edentulism) [3]; precision and confidence levels were 5.0 and 95%, respectively. The minimum sample size for the study was 198 older adults plus an additional 60.0% (*n* = 119) to compensate for potential non-participants previously described for populations of this type. Non-participants include those who refused to participate or were unreachable, institutionalized or deceased [9].

**Fig. 1:**
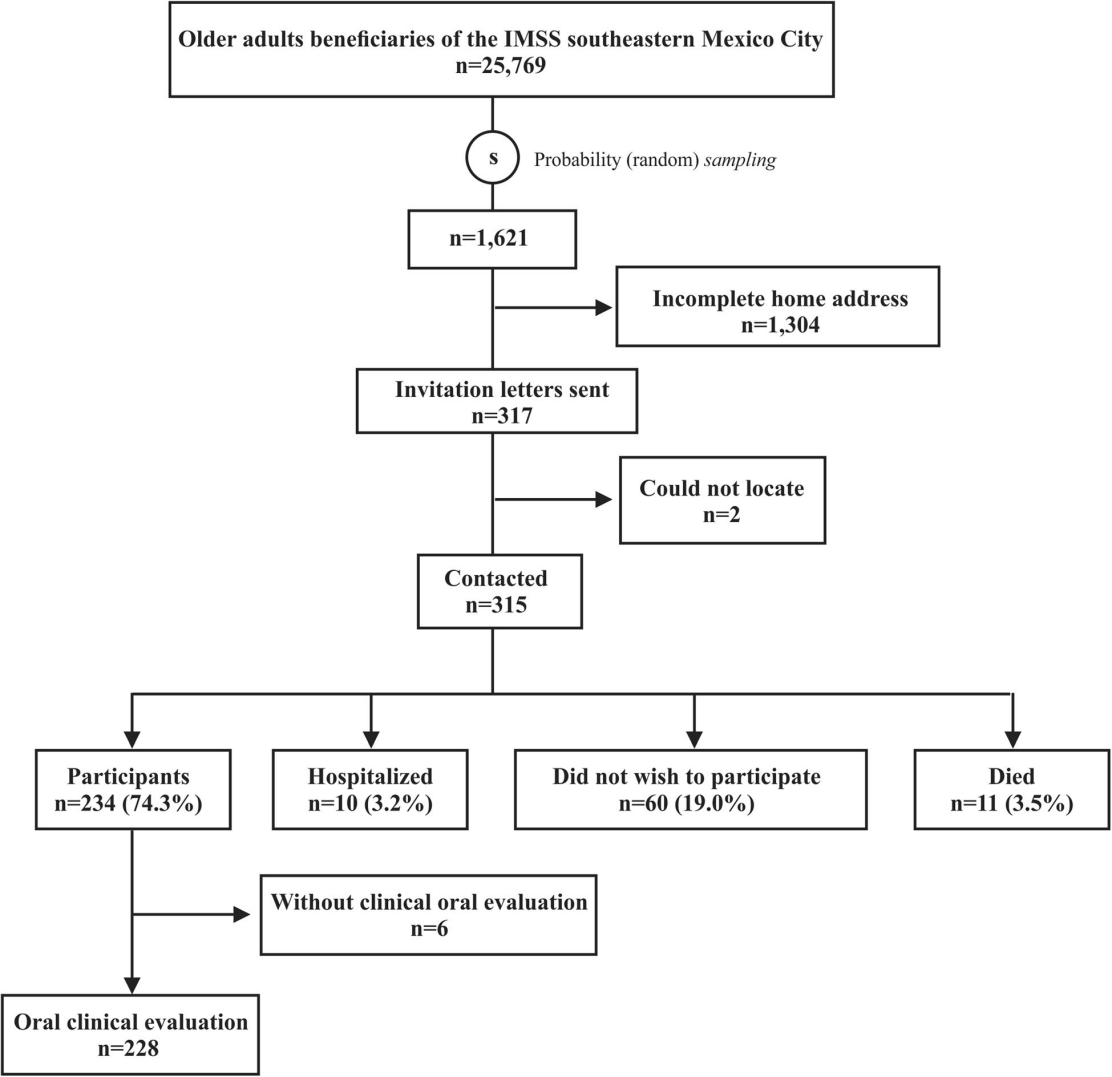
Composition of the sample of older adults beneficiaries of the IMSS in southeastern Mexico City

The registry of the last year 2013 of beneficiaries ≥60 years of age was available (*n* = 25,769) of one Family Medicine Unit (FMU) in southeastern Mexico City. A random selection was made to obtain 1,621 records to locate addresses and telephone numbers. It was noted that 80.4% (*n* = 1,304) of the records did not have a complete home address. There were 317 letters sent to the addresses of the older adults to inform them of the nature of the study and invite them to participate, as well as to provide them with the address of the FMU, day and time when they should present for the survey and corresponding clinical evaluation in case they wished to participate in the study. They were also provided with the telephone number where they could request further information and change their appointment if they so desired (Fig. 1).

Operating under the Mexican System of Social Security, the IMSS affords protection to salaried workers and their families not only in the form of medical and dental services but also economically, through disability and retirement pensions. In Mexico City, 48 FMUs offer primary-care services. The IMSS covers 36.5% of the general population and approximately 50.9% of older adults in Mexico City [10].

Our study protocol was reviewed and approved by the Local Committee for Research and Ethics in Health Research (No. 3609) of the Regional General Hospital No. 1, of Mexico City (Registration No. R-2014-3609-14).

### Data collection

We sent 317 letters to the homes of older adults inviting them to participate in the study, explaining what their involvement would consist of and specifying the date and time they should be present at the FMU. A telephone number was provided for those who needed to change their appointments. We subsequently called the recipients to remind them of their visits and arranged new schedules for those who could not attend as planned. During the appointments, we informed the older adults and their companions (where applicable) about the details of the study; those who agreed to participate were asked to sign an informed letter of consent.

Interviews were administered face to face to participants to ascertain their socio-demographic characteristics (age, sex, marital status, housing arrangements – specifically whether they lived alone - educational level and salaried work status). We also explored the presence of comorbidity and assessed cognitive function through the Mini-Mental State Examination (MMSE), adjusting for schooling; the cut-off score was set at ≤23 [11, 12]. We identified the presence of depression through the Center for Epidemiologic Studies Depression Scale-Revised (CESD-R): Major depression was determined where subjects had presented at least five symptoms necessarily including dysphoria (depressed mood) and/or anhedonia (inability to experience pleasure) for a minimum of 2 weeks together with three of the following symptoms: drastic weight loss/gain (appetite), sleep disorder, psychomotor agitation or retardation, fatigue, excessive or inappropriate guilt and suicide ideation [13, 14]. We also inquired whether they had used any medical or dental services in the last 12 months.

### Oral health-related quality of life (OHRQoL)

We assessed OHRQoL using the GOHAI-Sp. Validated in the Mexican older adult population [8], this instrument evaluates three dimensions of OHRQoL: (1) physical function, including eating, speaking and swallowing (Items 1, 2, 3 and 4); (2) psychosocial function, including concern about oral health, dissatisfaction with appearance, self-awareness in relation to oral health and difficulties in making social contact owing to dental problems (Items 6, 7, 9, 10 and 11); and (3) pain and discomfort requiring use of pharmaceutical products to alleviate soreness in the oral cavity (Items 5, 8 and 12). The GOHAI contains 12 Likert-scale items (two positives and 10 negatives) with five possible responses rated as follows: 1 = always, 2 = frequently, 3 = sometimes, 4 = hardly ever and 5 = never. Items 3 and 4 are rated inversely vis-à-vis the others, with conversion occurring at the time of data processing. The three GOHAI dimensions are graded by adding up the responses of interviewees: the higher the score, the higher the OHRQoL level. For our study, we considered the following dimensions: low physical function, low psychosocial function, pain and discomfort and low OHRQoL (corresponding to the GOHAI-Sp 25th percentile).

### Oral clinical evaluation

Two oral health professionals performed a clinical evaluation of oral health deficits in participating in older adults. They were specifically trained to determine the presence of painful chewing, dry mouth, xerostomia, plaque, calculus, coronal and root caries, tooth loss and gingival bleeding. With regard to the latter four, the evaluators presented intra- and inter-observer agreement levels of Kappa ≥0.80.

We defined total edentulism as the complete absence of all-natural teeth, and determined the presence of painful chewing where respondents replied affirmatively to the following question: “Have you ever felt discomfort or pain in your mouth when eating or chewing food of any kind?” Partial/full dentures were considered not functional on the bases of four parameters: stability, retention, extension and occlusion [15]. We established the presence of hyposalivation where participants took ≥4 min to dissolve a wafer in their mouths [16] and xerostomia where they scored ≥28 points under the Xerostomia Inventory [17]. To estimate plaque and calculus, the evaluators applied the Oral Hygiene Index [18]. They examined each tooth and registered the presence/absence of plaque according to Index parameters. Finally, they followed the World Health Organization criteria to determine coronal and root caries, tooth loss by a number of teeth per person and gingival bleeding per person [19].

### Statistical analysis

We used descriptive statistics to obtain frequencies, means, standard deviations (SDs) and medians (25th–75th percentiles). The Chi-squared test or Fisher’s exact test (if the expected number was less than five) was used to ascertain whether the frequencies and distributions were homogeneous. When comparing the means of more than two groups, we used variance analysis (ANOVA). The level of significance for the statistical tests was *p* < .05. When statistical differences were found, we used the Bonferroni post-hoc test to establish between which groups the difference occurred.

We used Mplus software version 5 to conduct Latent Class Analysis (LCA). LCA is a person/patient-centered approach used to classify individuals into mutually exclusive groups based on patterns of oral health deficiencies [3, 20]. We chose the number of classes for the model by progressively increasing the number and contrasting these results with those of each succeeding model using the Lo-Mendell-Rubin (LMR) likelihood ratio test, where the presence of a statistically insignificant probability (*p* > .05) suggests that the previous model (with a smaller number of classes) is preferable [20, 21].

We evaluated the goodness of fit of the model using a combination of the Akaike Information Criterion (AIC) [22] and the Bayesian Information Criterion (BIC) [23] as well as entropy values to obtain additional information regarding model fit [20]. Although no standard threshold exists for evaluating entropy, values close to 1.0 are considered the most desirable [3, 20, 21]. Finally, we interpreted the models in terms of their theoretical and practical coherence, choosing the simplest and most parsimonious model possible.

For the LCA, we considered the following oral health deficiencies: painful chewing, use of dentures, hyposalivation, xerostomia, plaque and calculus buildup, coronal and root caries, tooth loss and gingival bleeding. We used a percentile of ≥75th of gingival bleeding as the cut-off point for plaque, calculus, coronal and root caries and tooth loss [3].

We determined the strength of association (Odds Ratio [OR] and 95% confidence interval [95% CI]) through logistic regression between the latent classes (independent variables) and OHRoL dimensions in older adults (dependent variable). Finally, we adjusted the strength of association between the oral health categories and low OHRQoL for the other variables included in the study: age, sex, marital status, living arrangements (lives alone), educational level, paid work status, comorbidity, cognitive deterioration, depression and use of medical and dental services in the previous 12 months. Analyses were conducted with IBM-SPSS statistical package for Windows, Version 23.0 (IBM Corp. Released 2015. Armonk, NY: IBM Corp.).

## Results

We contacted 99.4% (*n* = 315) of the 317 older adults who were invited to participate in the study. Among these, 74.3% (*n* = 234) agreed to participate in the study by written informed consent, while 19.0% (*n* = 60) chose not to participate, 3.2% (*n* = 10) were hospitalized and 3.5% (*n* = 11) had died (Fig. 1).

We conducted interviews and oral clinical evaluations of 97.4% (*n* = 228) of the 234 older adults who agreed to participate. Six (2.6%) individuals, unwilling to undergo the clinical oral evaluation, were excluded from the study. The mean (SD) age of the remaining 228 participants was 68.4 (7.1) years. Women and men represented 64.0% (*n* = 146) and 36.0% (*n* = 82) of our sample, with mean ages of 69.1 (7.9) and 67.2 (5.0) (Table 1), respectively.

**Table 1:**
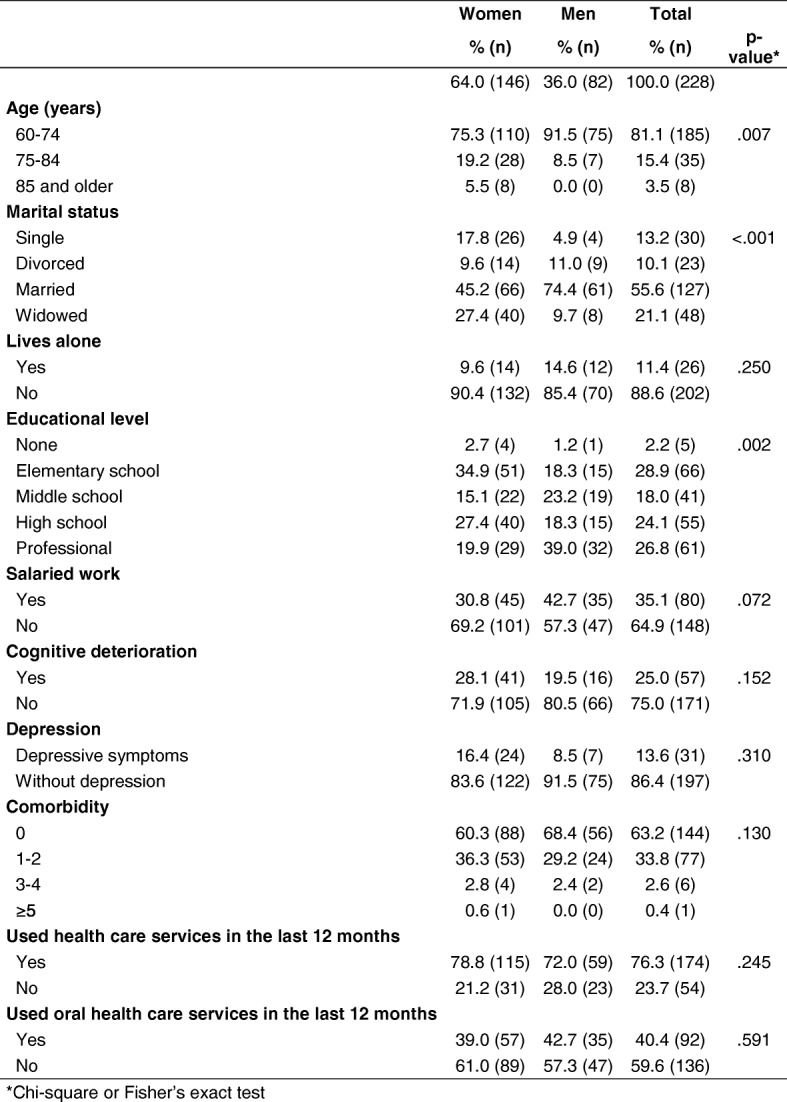
Characteristics of the study sample of older adults

*Chi-square or Fisher’s exact test

Concerning the dimensional results of OHRQoL, mean (SD) physical function was 16.2 (3.5) with a median (25th–75th percentile) of 11.0 (7.0–14.0); mean psychosocial function was 19.2 (3.9) with a median of 12.0 (9.0–15.0); and mean pain and discomfort came to 12.0 (2.7) with a median of 7.0 (6.0–11.0). The GOHAI yielded a mean of 47.5 (8.7) and a median of 31.0 (24.0–39.0). As regards oral health variables, we found that 9.6% (*n* = 22) of participants were edentulous, 84.2% (*n* = 192) were partially edentulous and 6.1% (*n* = 14) had 28 teeth. Of the 22 edentulous older adults, 86.4% (*n* = 19) used dentures, while 42.2% (*n* = 81) of the partially edentulous older adults did so. All of the dentures were evaluated and found to be functional. Table 2 shows oral health deficits in the study sample of older adults.

**Table 2:**
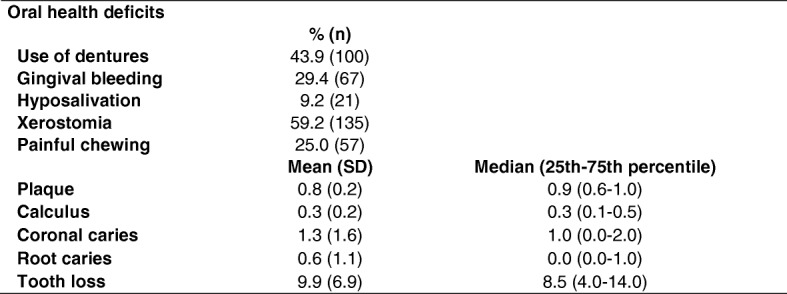
Oral health deficits in of the study sample of older adults

Table 3 shows the goodness of fit of the model and the conditional probability associated with the latent classes of oral health. Under LCA, three was the optimum number of classes for categorizing oral health in older adults, taking into account goodness of fit (AIC = 2398.643 and BIC = 2505.135), entropy (0.805) and the LMR likelihood ratio test results (*p* = .048).

**Table 3:**
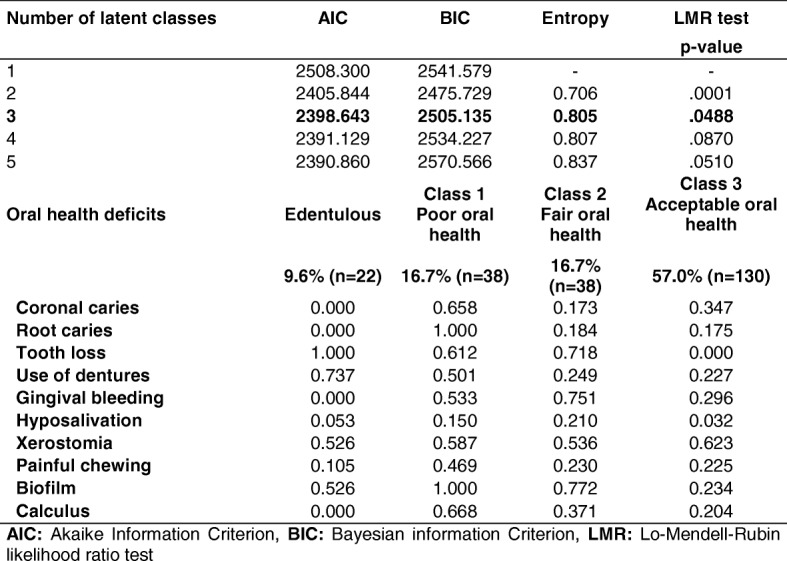
Goodness of fit of the latent class model and conditional probability associated with oral health

*AIC* Akaike Information Criterion, *BIC* Bayesian information Criterion, *LMR* Lo-Mendell-Rubin likelihood ratio test

**Fig. 2:**
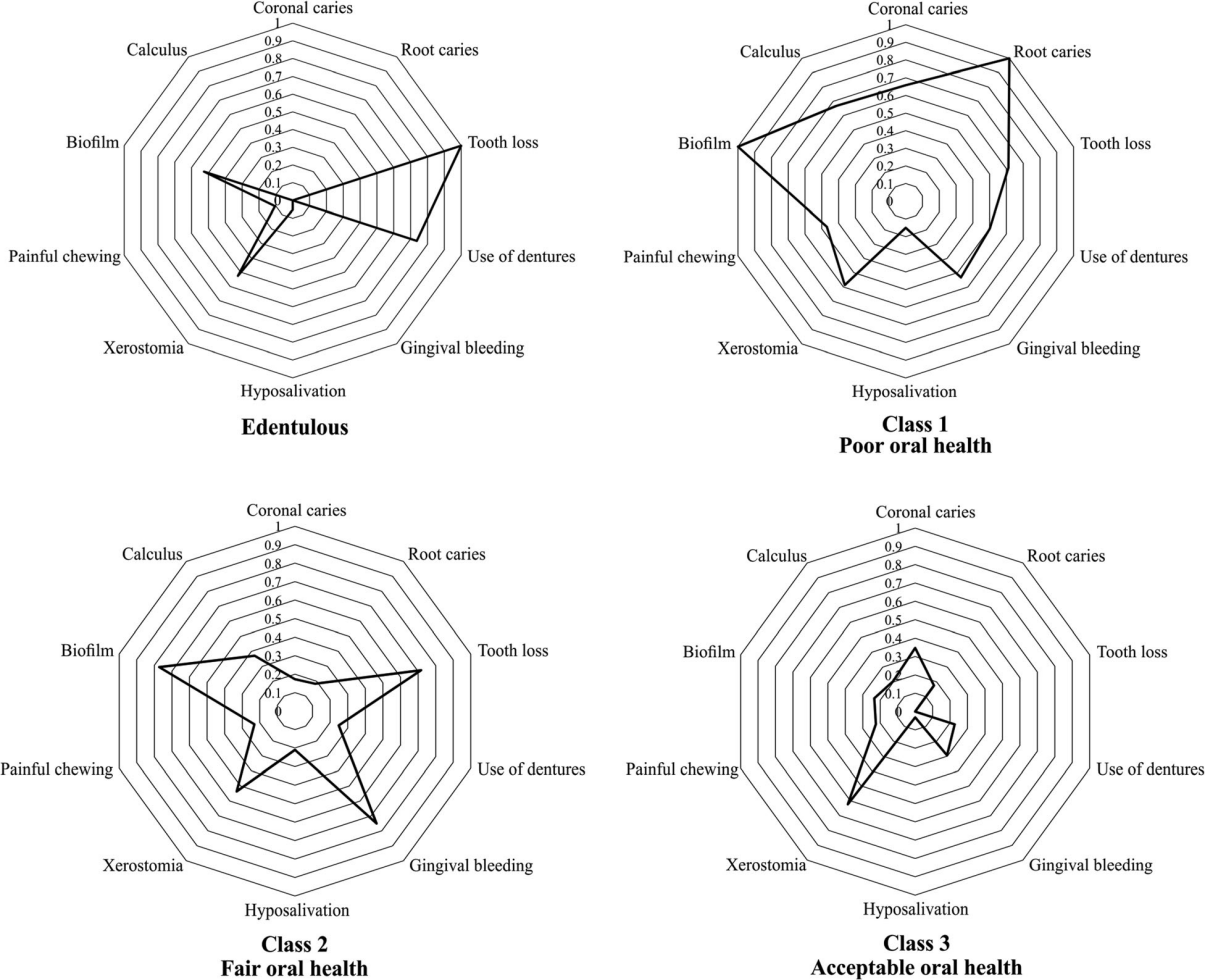
Latent class for oral health in older adults

Among our total sample of older adults, 57.0% (*n* = 130) demonstrated poor (Class 1), 16.7% (*n* = 38) fair (Class 2) and 16.7% (*n* = 38) acceptable oral health (Class 3) (Fig. 2).

Table 4 shows the mean (SD) scores of the GOHAI dimensions for the three oral health classes and the edentulous older adults. With regard to physical function, a statistically significant difference was observed between the mean for older adults with acceptable oral health (Class 3) and the mean for those with poor oral health (Class 1), fair oral health (Class 2) and edentulous status. As regards the psychosocial function, a difference existed between the mean of older adults with acceptable oral health (Class 3) and the mean of those with poor (Class 1) and fair oral health (Class 2); as well as between the mean of those with poor oral health (Class 1) and edentulous status. Concerning pain and discomfort, we found statistically significant differences between the mean scores of older adults with acceptable oral health (Class 3) and those with poor oral health (Class 1). Finally, statistically significant differences emerged in the GOHAI between the mean scores of older adults with acceptable oral health (Class 3) and those of older adults with poor (Class 1) and fair (Class 2) oral health, but not between the means of the first and the edentulous participants.

**Table 4:**
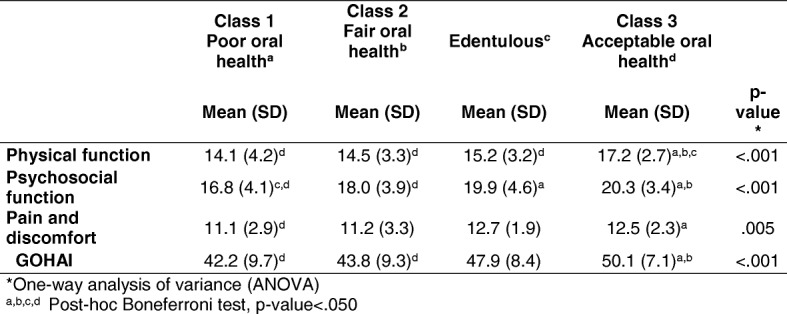
Mean (SD) score of GOHAI dimensions for edentulous and the three oral health classes in older adults

*One-way analysis of variance (ANOVA)

a, b, c, d Post-hoc Boneferroni test, *p*-value<.050

Table 5 shows the crude and adjusted strength of association (OR, 95% CI) between older adults with oral health category (latent classes) and edentulous status on one hand, and on the other, those with a low OHRQoL in physical and psychosocial function, pain and discomfort, and the GOHAI. With Class 3 (adults with acceptable oral health) established as a reference, older adults categorized as having poor oral health (Class 1) demonstrated a stronger strength of association (OR and adjusted OR) for exhibiting low OHRQoL in the areas of physical and psychosocial function as well as in the GOHAI. Older adults categorized as having fair oral health (Class 2) showed a stronger strength of association for suffering from pain and discomfort.

**Table 5:**
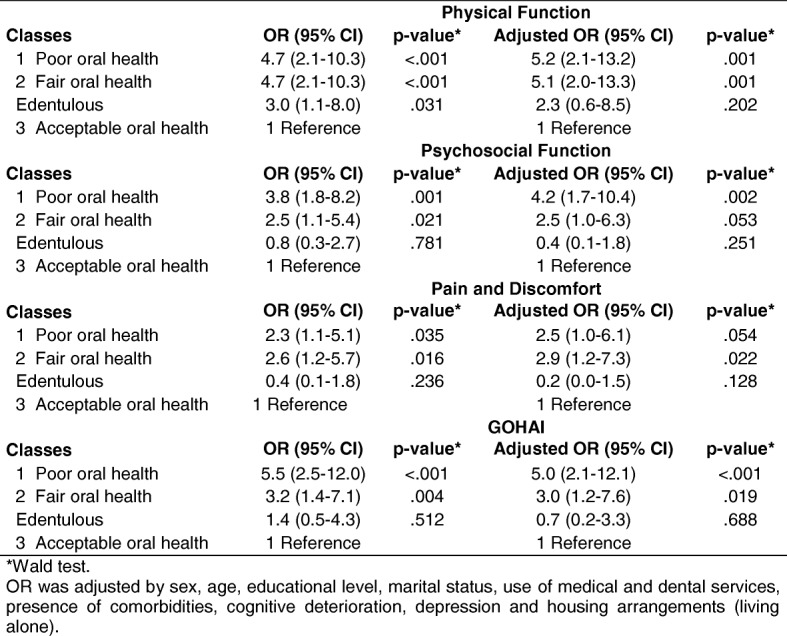
Strength of association between low OHRQoL and the oral health classes in older adults

Wald test

OR was adjusted by sex, age, educational level, marital status, use of medical and dental services, presence of comorbidities, cognitive deterioration, depression and housing arrangements (living alone)

## Discussion

Oral health problems begin at an early age and become a greater problem at advanced ages. Such is the case with tooth loss, edentulism, clinical attachment loss, coronal and root caries, oral mucosal lesions, use of nonfunctional dental prostheses (partial or total), chewing problems, among other conditions [4].

Traditionally, the oral health of the population, and particularly in older adults, has been limited to clinical indicators and oral indexes, as well as to the presence or absence of a separate disease [1] that is, each factor separately contributes information on each oral health component, and thus the individuals are not classified based on the patterns of the oral health components. The ability to relate oral health and quality of life in older adults represented a challenge due to the multifactorial condition of both concepts. The results of our study suggest that poor oral health is associated with poor oral OHRQoL, confirming the results of other studies [24–26]. We found that community-dwelling older adults suffering from poor oral health also exhibited low physical and psychosocial function as well as pain and discomfort. They also scored lower on the GOHAI. This carries important consequences for their ability to perform daily activities and implies problems in their oral physiological functioning and social communication [27]. For this reason, a redefinition of health policies should be considered in the near future, as the consequences of poor oral health could lead to low quality of life and increased health care costs [3].

In older adults, we see signs of the health habits acquired in the first stages of life, bearing in mind that the aging process generates a series of changes in social status, sensory perception and cognitive and motor functions. In general, the social background is closely related to the risk of illnesses such as caries and periodontal disease. The social context exerts a powerful influence on individual behavior, with oral hygiene emerging as the most important behavioral factor identified [28]. At IMSS, oral health services are limited to prevention and control treatments, as well as restoration with amalgam or composite resin, and dental extractions. All restoration and rehabilitation treatments must be covered by private oral health services, so it is possible that the population of older adults without social security and may have even worse oral health than our participants [3].

Tooth loss is one of the principal elements that can affect the biopsychosocial condition of individuals. It has been recognized that tooth loss is not solely the result of untreated caries or the consequence of periodontal disease; it may also result from traumas, indications for the extraction of teeth in orthodontic procedure and a set of other complex factors not directly related to dental diseases, such as unfavorable attitudes towards health care, lack of access to oral health services and reluctance or inability to pay [28]. Our results show that the OHRQoL in edentulous older adults is similar to that exhibited by older adults with acceptable oral health. In this study, 100% of those using full dentures had functional prostheses, leading to the conclusion that adequate prosthetic rehabilitation allows older adults to perform their functional, physical and psychosocial activities without pain or discomfort and therefore with positive results in their OHRQoL [29]. This does not mean that older adults who have few teeth in mouths, need to extract their remanent teeth to rehabilitate their mouths with prostheses, by the close with adequate partial dentures can also effective for functional, physical and psychosocial activities and improve quality of life. Nevertheless, an aging population is experimenting less edentulism and greater tooth retention, because, older adults have more and better regular oral health care and prevention services that improve their quality of life [30, 31].

According to LCA, a methodology for determining the typology of oral health, we considered three classes of oral health related to the parameters studied [4, 21, 22] and observed a discrimination of the classes relative to OHRQoL: older adults with acceptable oral health obtained higher OHRQoL scores, followed by those with fair oral health and finally by those with poor oral health. Accordingly, LCA can be considered a useful tool for determining typology in other studies needing to classify older adults according to the state of their oral health [32].

Our study was limited in that it did not consider the evolution of the exposure-effect association over time and was therefore unable to establish the possible causal relationship between oral health and OHRQoL in the observed population. A longitudinal design would be more effective for measuring the impact and magnitude of the independent variable in time and thus for establishing the causal relationship between oral health and OHRQoL among community-dwelling older adults. On the other hand, the lack of studies using this methodology of latent class analysis to determine the typology of oral health in older adults makes it impossible for us to make a.

## Conclusions

The results of our study suggest that poor oral health is associated with low OHRQoL in older adults. It is therefore essential to design and implement oral health care policies specifically targeted at improving the quality of life in this older adult population.

## Data Availability

Data is available upon request. Contact e-mail: sergio.sanchezga@imss.gob.mx

## Abbreviations

AIC: Akaike Information Criterion
BIC: Bayesian Information Criterion
CESD-R: Center for Epidemiologic Studies Depression Scale-Revised
FMU: Family Medicine Unit
GOHAI: Geriatric Oral Health Assessment Index
IMSS: Mexican Institute of Social Security
LCA: Latent Class Analysis
LMR: Lo-Mendell-Rubin
MMSE: Mini-Mental State Examination
OHRQoL: Oral Health on the oral health-related Quality of Life
